# From theory to practice in training health researchers in patient and public involvement: a scoping review protocol

**DOI:** 10.1186/s13643-025-02966-1

**Published:** 2025-12-03

**Authors:** Caline Jesus, Isabelly Cristina Rodrigues Regalado, Karolinne Souza Monteiro, Adriana Gomes Magalhães, Paula Silva de Carvalho Chagas, Christina D. C. M. Faria, Vanessa Vega Córdova, Izaskun Álvarez-Aguado, Eve Namisango, Christopher Morris, Egmar Longo

**Affiliations:** 1 Graduate Program in Decision and Health Models, Universidade Federal da Paraíba, João Pessoa, Brazil; 2 Graduate Program in Physiotherapy, Universidade Federal Do Rio Grande Do Norte, Natal, Brazil; 3 Graduate Program in Rehabilitation Sciences and Physical and -Functional Performance, Universidade Federal de Juiz de Fora, Juiz de Fora, Brazil; 4 Department of Physical Therapy, Universidade Federal de Minas Gerais, Belo Horizonte, Brazil; 5 Pontificia Universidad Católica de Valparaíso, Valparaíso, Chile; 6 Universidad de Las Américas, Providencia, Chile; 7 African Palliative Care Association, Kampala, Uganda; 8 Peninsula Childhood Disability Research Unit (PenCRU) and NIHR Applied Research Collaboration South West Peninsula (PenARC), University of Exeter Medical School, University of Exeter, Exeter, UK

**Keywords:** Patient and public involvement, Patient engagement, Patient participation, Research training, PPI training, Health researchers, Doctoral researchers, Scoping review

## Abstract

**Background:**

Patient and public involvement (PPI) aims to increase the relevance and impact of research by ensuring that outcomes align with the real needs of those involved. In PPI, the public actively participates in all stages of research, which has been shown to improve research quality, empower participants, and enrich researchers’ understanding of patients’ and the public’s lived experiences. However, implementing PPI poses a challenge for many researchers, making training essential as it provides the necessary skills to incorporate PPI meaningfully into their projects. Thus, this review aims to map existing PPI training programs in health research, identifying effective strategies that can be replicated, thereby contributing to the improvement of health research practices.

**Methods:**

The scoping review will adhere to Arksey and O’Malley’s six-step framework and the PRISMA Extension for Scoping Reviews (PRISMA-ScR) checklist, with results reported following the GRIPP2—Short Form. This review will follow the JBI guidelines for scoping reviews. A PPI group will be established to contribute to the interpretation and discussion of findings and will also be recognized as co-authors of the article. Searches will be conducted in MEDLINE (Ovid), Embase (Elsevier), EBM Reviews, CINAHL (EBSCO), Scopus, ERIC (EBSCO/ProQuest), Web of Science Core, HealthSTAR (Ovid), and Academic Search Complete. Gray literature searches will be conducted in ASSIA (ProQuest) and Google Scholar. The review will include full-text articles published with no language restrictions, focusing on PPI training programs for health researchers, with no publication date restrictions. Two independent reviewers will screen studies by title and abstract, followed by full-text review.

**Discussion:**

The study aims to identify and systematize the approaches used in PPI training programs, providing a foundation to guide researchers in effective PPI implementation. This review may encourage health researchers to integrate PPI as an essential component throughout the research cycle, aligning studies more closely with patients’ needs and experiences. Such alignment has the potential to directly inform public health policies, benefiting the target population.

**Systematic review registration:**

10.17605/OSF.IO/WBDPE.

## Background

International recognition highlights that the active involvement of patients, caregivers, and community members in planning, conducting, and disseminating research enhances its relevance, quality, and impact [1–4]. Evidence from previous reviews demonstrates positive impacts, including improved research quality and appropriateness [5], empowerment of service users [2], and increased researcher awareness of patients’ lived experiences [2, 6].

The term patient and public involvement (PPI) refers to the practice of engaging patients and members of the public in the research process not merely as participants, but as active partners. We define public involvement in research as an investigation carried out *with* or *by* members of the public, rather than *for*, *about*, or *on* them. In this way, research becomes a shared responsibility rather than the exclusive domain of researchers and professionals [7].

**Fig. 1:**
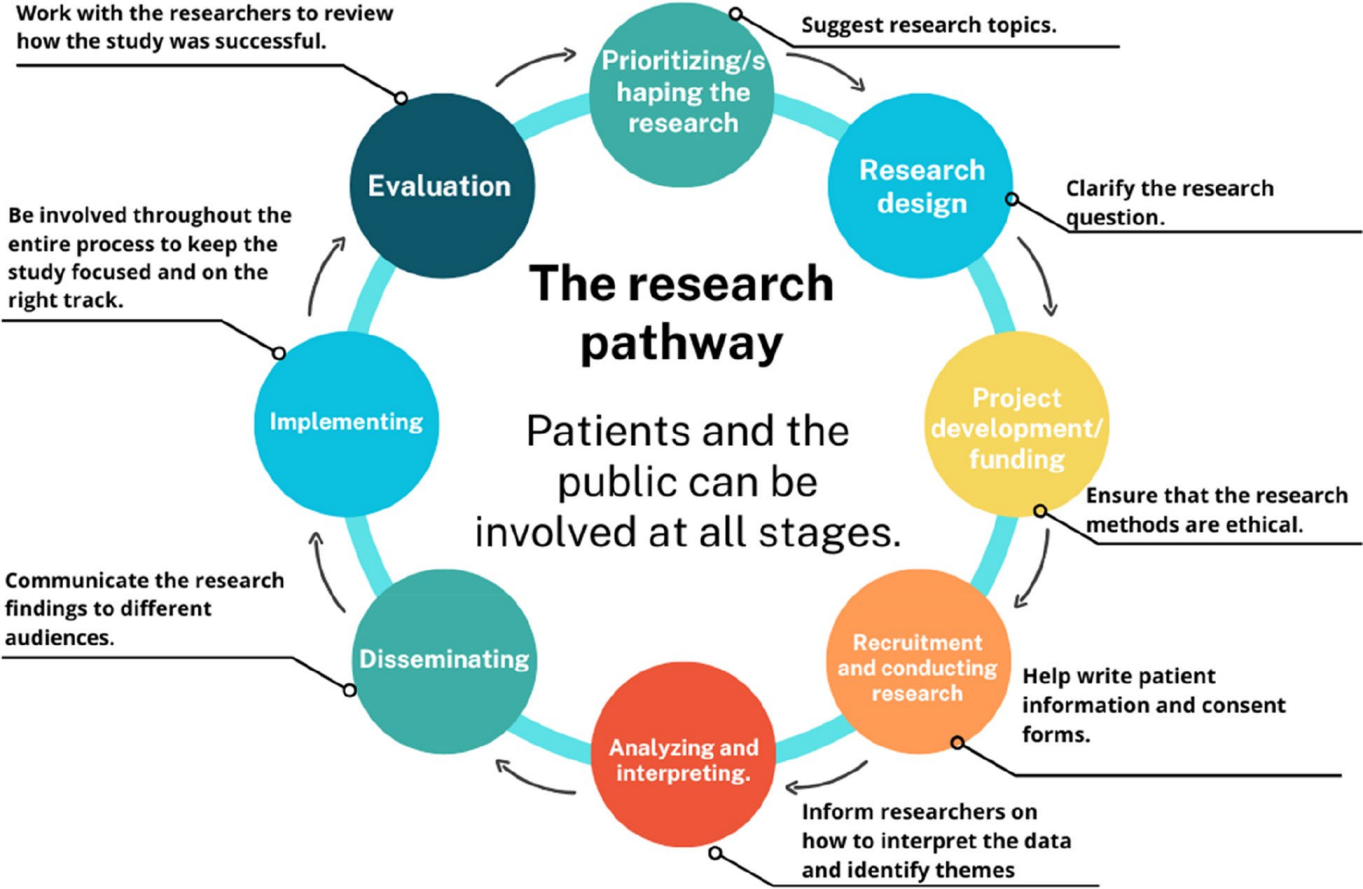
Ways for the public to get involved in research. Source: adapted by the authors based on NIHR

PPI can involve the public throughout the research pathway, from identifying research priorities to disseminating findings (Fig. 1). The term “*public*” encompasses patients, potential patients, caregivers, users of health and social care services, and representatives of organizations that use these services [3].

PPI recognizes the importance of public perspectives and concerns, emphasizing that incorporating these voices into research enhances the effectiveness and value of health research [3] and has the potential to improve research design and conduct [8, 9]. Core values associated with PPI include equality, respect, and transparency between researchers and PPI partners [10].

The public can contribute unique insights to health research by enhancing its quality, transparency, and relevance to patients; improving recruitment and retention rates; broadening the diversity of study participants; and strengthening the dissemination of findings beyond the academic community [11, 12].

PPI in health research is already well established in many countries, with several models and theoretical frameworks developed to support its implementation. Examples include the Patient-Centered Outcomes Research Institute (PCORI) in the United States of America (USA) [13], the Strategy for Patient-Oriented Research (SPOR) in Canada [14], the Consumers Health Forum (CHF) in Australia [15], and the National Institute for Health and Care Research (NIHR) in the United Kingdom (UK) [3].

However, several studies show that implementing PPI in practice is not straightforward [16, 17]. To strengthen stakeholder engagement, researchers must plan diverse forms of involvement, allocate resources effectively, provide ongoing training, and ensure active participation of stakeholders [18]. In other words, beyond research competencies, researchers conducting patient-involved studies need to know how to engage patients, manage project logistics, and collaborate with others in shaping research directions. This requires communication, interpersonal, managerial, and financial skills in addition to scientific expertise [19].

PPI is rarely included in the core curriculum for researchers, creating multiple challenges when they attempt to involve patients and the public. Many lack knowledge of PPI concepts and may rely on personal interpretations of how to apply them in practice [18, 20]. Effectively involving patients and the public in the research process is a complex competency that requires practice and training to demonstrate the benefits of PPI and to provide guidance on how to implement it across different types of projects [20, 21].

PPI training has the potential to directly influence how PPI is understood and implemented, but it requires an investment of time and resources [22, 23]. Studies show that PPI training increases researchers’ awareness and understanding of the value and importance of PPI in conducting studies [23]. Such training ensures that researchers acquire the necessary skills to integrate PPI into the research process [22, 23] and provides the confidence required to conduct PPI effectively [22, 24].

PPI training can be described as any activity “*aimed at helping public members and researchers develop their knowledge, skills, and experience, preparing them for public involvement in research*” [25]. Some educational programs and resources on PPI already exist. An example is provided by NIHR Applied Research Collaboration (ARC) West, which offers information on its website about training and capacity-building opportunities for researchers, healthcare professionals, and other stakeholders [26]. Another example is the extensive PPI workshop program implemented by the University College London Hospitals (UCLH) Biomedical Research Centre (BRC), targeting researchers and integrated into its strategy to strengthen a PPI culture in research. The program aims to develop practical skills and increase researchers’ confidence in working with patients and the public, and it is continuously reviewed and adapted to meet participants’ needs [24].

Aries et al. suggest that PPI should be carefully planned as a core component from the outset of a research project. The aim is to achieve early engagement and full integration of at least one patient or public member at each stage of the project. They also recommend practices such as sensitive task selection, appropriate compensation for PPI advisors, promotion of transparent and respectful communication, and provision of meaningful PPI training for all involved parties [26].

A lack of researcher knowledge about the benefits and risks of using this research model, as well as its impact on outcomes, is one reason why the most appropriate PPI methods are not utilized. Other factors limiting researchers’ use of PPI include reluctance to share control over the research agenda, resistance to change, time pressures, and tokenism [20].

In health research, effective interaction with patients and the public is a critical aspect that plays a key role in enhancing research excellence and ensuring the relevance and applicability of findings. However, the ability to engage stakeholders meaningfully and productively in the research process remains a challenge. Despite growing recognition of PPI in research, studies addressing training in this area have been limited [22, 23].

Despite growing recognition of the value of PPI in health research, guidance on training health researchers to implement PPI effectively remains limited. No comprehensive synthesis currently maps the available PPI training programs. This scoping review aims to provide a comprehensive overview of existing PPI training programs in health research. Specifically, it seeks to identify and describe these programs, examine the contexts in which they are implemented, and evaluate their reported outcomes and effectiveness.

## Methods

### Study type

This scoping review will use the Joanna Briggs Institute (JBI) methodology proposed by the JBI Reviewers Manual [27]. Scoping reviews are widely used to map the breadth and depth of knowledge on a specific topic. This methodology has been rigorously applied to collect, evaluate, and present existing research findings, allowing for comprehensive model building and expanding the possibilities for explaining the findings. We adopted the step-by-step structure of the scoping review by Arksey and O’Malley [28] to guide this review. The current review will be reported according to the PRISMA Extension for Scoping Reviews (PRISMA-ScR) [29], and its results will be reported considering the Guidance for Reporting Involvement of Patients and the Public—Short Form (GRIPP2-SF) [30].

### PPI group

A PPI group, comprising patients, clinicians, and researchers from across Brazil, was established to oversee and contribute to the review. Recruitment was conducted through social media announcements and invitations to individuals already recognized as patient advocates in the country. To ensure diversity and representativeness, the group includes members from all regions of Brazil.

Given that PPI is still in an early stage of development in Brazil, and that some members lack formal training in scoping review methods or PPI research, their role will focus primarily on the analysis and interpretation of findings, as well as the dissemination of results, ensuring that outputs are relevant, accessible, and meaningful to diverse audiences.

Meetings will be held biweekly, lasting approximately 1 h, to collect input and support shared decision-making. Contributions from the group will be systematically documented, including a record of changes and decisions made, and feedback will be incorporated into the review as appropriate. Activities and contributions of the PPI group will be reported in accordance with the GRIPP2-SF checklist, ensuring transparency regarding participant involvement in the review. Members will be recognized as co-authors whenever authorship criteria are met.

The participation of the PPI group in this review has been approved by the Research Ethics Committee of the Federal University of Paraíba (UFPB) (CAAE: 79883924.2.1001.5188 and CAAE: 88570725.4.0000.5188).

#### Step 1: identifying the research question

This scoping review is guided by the following research questions:

1. What are the key characteristics of PPI training programs for health researchers?
2. In which contexts are these PPI training programs implemented?
3. What outcomes and evidence of effectiveness are reported from these training programs?

The Population, Concept, and Context (PCC) mnemonic was employed to formulate the research question, following JBI guidelines for scoping reviews. Through this technique, the guiding question for this review was: “What are the PPI training programs for health researchers?”

Population: For this review, the population will refer to “health research professionals,” including physicians, PhD holders, and researchers involved in studies that integrate PPI training into their projects. If a study considered for inclusion does not clearly specify whether the population involved meets the inclusion criteria, additional information will be sought (e.g., contacting study authors, consulting supplementary materials, or checking trial registries).

For this review, doctoral students, early career researchers, and clinicians are a particularly important group as they are conventionally aligned with preclinical research either as clinician-researchers or steering research to ensure the translation of findings is possible [27].

Concept: PPI as defined by the NIHR (1). This includes PPI approaches used at any point in the research cycle involving patients and/or the public; however, there are variations in how the terms are used internationally. Therefore, the following terms will be included: “patient engagement,” “patient and public involvement,” “patient and public engagement,” “public involvement,” “participatory research,” “patient involvement,” “consumer engagement,” “community engagement,” “public deliberation,” “deliberation,” “community involvement,” “community advisory board,” “steering committee,” “community-driven research,” “community-engaged research,” “participatory action research,” “community-based participatory research,” “community-oriented,” “stakeholder advisory panel,” “patient and stakeholder engagement.”

Context: Relevant publications involving terms such as “PhD program,” “Workshop,” and “Training” will be included. No language restrictions will be applied.

### Inclusion and exclusion criteria

Articles will be selected based on the following inclusion criteria: (1) reporting training programs in PPI; (2) involving health researchers in the training programs; (3) no language restrictions, with multilingual synonyms of key terms included to ensure comprehensive coverage; (4) including all records from the inception of each database, with no publication date restrictions; and (5) considering manuscripts regardless of publication status or source (e.g., peer-reviewed journal articles, gray literature). No limitations on study design will be imposed. Secondary sources (e.g., systematic literature reviews, narrative reviews) will be excluded.

#### Step 2: identifying relevant studies

The search strategy will be developed in consultation with an experienced librarian and will include the following databases: MEDLINE (Ovid), Embase (Elsevier), EBM Reviews, CINAHL (EBSCO), Scopus, ERIC (EBSCO/ProQuest), Web of Science Core, HealthSTAR (Ovid), and Academic Search Complete. Gray literature searches will be conducted in ASSIA (ProQuest) and Google Scholar, as well as on relevant websites and online platforms that provide PPI training materials, which will be systematically explored using Google. For Google Scholar, searches will be limited to the first 200 results per query, and the date of each search will be recorded to ensure transparency and reproducibility.

**Fig. 2:**
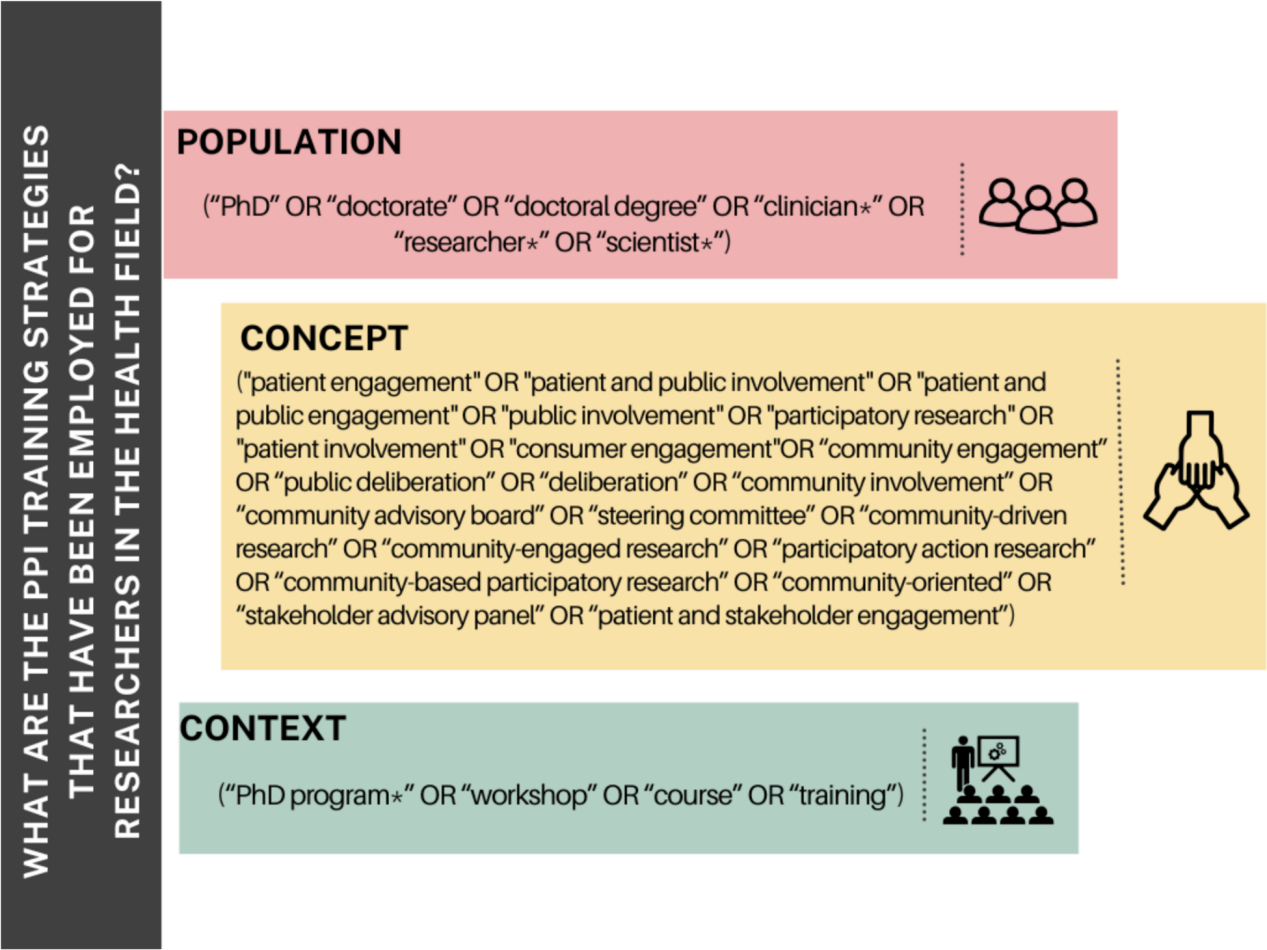
English keywords for database searches

**Fig. 3:**
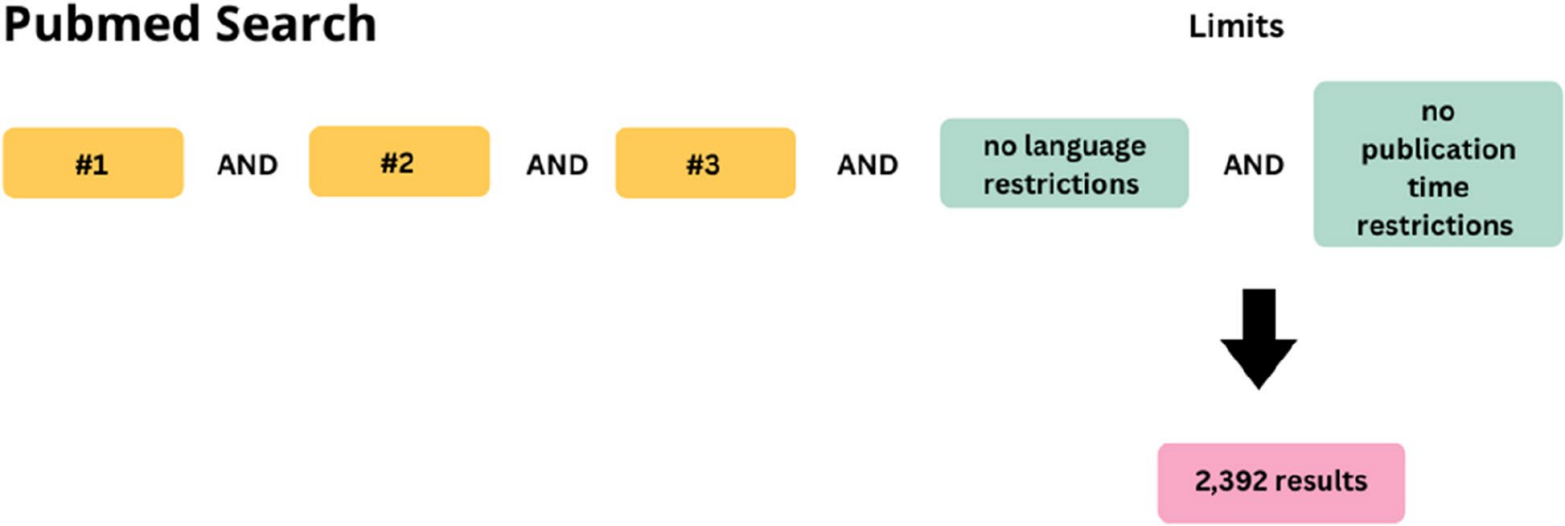
PubMed search strategy

The literature search will commence in November 2025, ensuring that the most recent and relevant studies are captured at the time of review. The following English keywords will be searched for in titles and abstracts; a complete list can be found in Fig. 2: “Clinicians,” “PhD,” “Researcher,” “Patient and public engagement,” “patient engagement,” “public involvement,” “participatory research,” “patient involvement,” “consumer engagement,” “PhD programme,” “workshop,” “Course,” “training.” The final search results will be exported into Zotero to remove duplicates. Then, the results will be exported to Rayyan (https://www.rayyan.ai/). An example of the search strategy used in PubMed is illustrated in Fig. 3, where #1 represents the population, #2 the concept, and #3 the context.

#### Step 3: study selection

After conducting the literature search and removing duplicates, the titles and abstracts of each study will be screened for eligibility according to the inclusion criteria outlined above. Studies that may meet the inclusion criteria will be retrieved in full. The review decision process will be presented in a PRISMA flowchart [28].

Before starting the full screening process, each reviewer will independently examine ten included studies before comparing the results to ensure consistency. Two researchers (CJ and KSM), using RAYYAN, will independently review the title and abstract of each record before meeting to compare their findings. Any discrepancies will be resolved through discussion or with the assistance of a third reviewer (EL) if consensus cannot be reached.

After title and abstract screening, full-text screening will be conducted using an iterative approach. Two reviewers will independently pilot screen a random sample of approximately 50 full-text articles to ensure consistency and refine the inclusion and exclusion criteria. Subsequently, all full texts will be screened independently by the two reviewers. Any disagreements will be resolved through discussion, with a third reviewer consulted if consensus cannot be reached [31].

#### Step 4: extraction and data charting

We will classify retrieved publications into research, theoretical/conceptual publications, and gray literature using a data extraction instrument developed for this study.

A data organization form will be developed in spreadsheet format, according to JBI guidelines [31]. Two reviewers will test the data extraction form using five articles to measure agreement. The form will include a mapping of the publications and will organize the extracted information according to the following categories:

1. Authors
2. Date of publication
3. Origin/country of origin (where the source was published or conducted)
4. Objectives/purposes of the study established
5. Population
6. Methodology/methods (design; people involved; stages of involvement; level or nature of involvement; qualitative evidence of impact; quantitative evidence of impact; measure robustness; economic evaluation; format of programs and/or training interventions, content taught, instructor(s), duration, purpose, training budget)
7. Type of intervention, comparator, and details (e.g., duration of the intervention) (if applicable)
8. Outcomes
  8.1. Primary outcomes: improvement in researcher knowledge, skills, and self-efficacy related to PPI
  8.2. Secondary outcomes: changes in research practices, improvement in the quality of PPI projects, acquisition of future grants or funding opportunities, barriers and facilitators to participating in PPI training

9. Key findings (impacts—contribution to new knowledge; context factors; process factors; reflections/critical perspective)
10. Gaps in existing research

Any discrepancies will be solved through discussion until consensus is reached or by including a third reviewer to act as an arbitrator. Once the accuracy and comprehensiveness of the tool are established, we will proceed with the complete data extraction.

#### Step 5: collating, summarizing and reporting the findings

This study aims to collect data on PPI training with the objective of mapping the different modes of training available worldwide. The PCC inclusion criteria will guide the data mapping. To collect, summarize, and report the results, we will adopt a mixed analytical approach, combining quantitative descriptive elements with qualitative thematic synthesis.

The quantitative descriptive analysis will include the nature and distribution of the studies, the characteristics of the training programs and/or interventions, and the contexts in which these programs and/or interventions are being implemented. Extracted data related to study characteristics will be tabulated, and a descriptive summary will accompany the results, illustrating how the findings address the research questions of this scoping review.

In addition, a qualitative thematic synthesis will be conducted to interpret and discuss the nature of the training programs, the implementation contexts, and the significant implications of each study. This synthesis will also allow us to identify gaps in the literature and highlight opportunities for future research, in consultation with the PPI group.

A PRISMA flow diagram will be produced to present the number of included studies and the reasons for full-text exclusions. By combining quantitative descriptive analysis with qualitative thematic synthesis, this approach appropriately reflects the mixed nature of the methodology and ensures greater clarity and transparency in reporting.

#### Step 6: consultation with the PPI group

Although the inclusion of stakeholders is optional [29], the establishment of a PPI group is a fundamental component of this scoping review and demonstrates our commitment to PPI in this project. Prior to the analysis, PPI members will receive a brief orientation on systematically reviewing extracted data. Subsequently, they will collaborate with the research team in reviewing the extracted data. Analytic rigor will be maintained through systematic documentation of decisions, triangulation of perspectives, and reflexive discussions. Authorship will be offered to PPI members who make substantial contributions to the interpretation of results and manuscript preparation, while other contributors will be acknowledged appropriately. The findings will inform strategies for training health researchers intending to implement PPI in their projects.

Publication and dissemination plan: Upon completion of the scoping review, the findings will be submitted for publication in a peer-reviewed journal focusing on health research or methodology. Key results will also be presented at national and international conferences. The research team will prepare a plain-language summary to share with the PPI group, ensuring that the findings are understandable to the general public and enabling group members to actively contribute to their dissemination. Additionally, results will be disseminated through institutional websites, social media platforms, and postgraduate programs to promote broader awareness and knowledge translation.

## Discussion

Through this scoping review, we will map the literature on training methods in PPI for health researchers. Our goal is to support health researchers who wish to effectively implement PPI in their projects. We aim to understand the contexts in which these training programs are implemented, which will reveal best practices and effective strategies for PPI implementation that can be replicated or adapted by researchers based on their specific needs. Involving the interest group in the discussion of these results will certainly enhance the relevance of this review.

The appropriate use of PPI in health research can directly influence public health policies, as this approach fosters active listening to the studied public, ensuring that their real needs are heard. We aim to contribute to those who wish to implement PPI effectively in their projects.

### Implications and limitations

This scoping review will provide a comprehensive overview of training programs on PPI for health researchers, mapping their components, contexts, and implementation strategies. The review will also identify gaps in the current evidence base that may guide the design of future training and inform policy and practice.

A key limitation is that only published studies will be included, which may introduce publication bias and restrict the comprehensiveness of the evidence base. To mitigate this risk, a broad range of databases will be searched, reference lists will be screened, and citation tracking will be conducted to maximize retrieval of relevant publications.

Future work should focus on evaluating the effectiveness of PPI training interventions and developing consensus on core components to strengthen their implementation across diverse health research settings.

## Data Availability

Not applicable.

## Abbreviations

PPI: Patient and public involvement
PRISMA-ScR: Preferred Reporting Items for Systematic Reviews and Meta-Analyses for Scoping Reviews
PCORI: Patient-Centered Outcomes Research Institute
SPOR: Strategy for Patient-Oriented Research
CHF: Consumers Health Forum
NIHR: National Institute for Health and Care Research
ARC: Applied Research Collaboration
JBI: Joanna Briggs Institute
GRIPP2-SF: Guidance for Reporting Involvement of Patients and the Public—Short Form
